# Appetitive traits as behavioural pathways in genetic susceptibility to obesity: a population-based cross-sectional study

**DOI:** 10.1038/srep14726

**Published:** 2015-10-01

**Authors:** Hanna Konttinen, Clare Llewellyn, Jane Wardle, Karri Silventoinen, Anni Joensuu, Satu Männistö, Veikko Salomaa, Pekka Jousilahti, Jaakko Kaprio, Markus Perola, Ari Haukkala

**Affiliations:** 1 Department of Social Research, University of Helsinki, Helsinki, Finland; 2 Department of Epidemiology and Public Health, University College London, London, UK; 3 Institute for Molecular Medicine Finland (FIMM), University of Helsinki, Helsinki, Finland; 4 Department of Health, National Institute for Health and Welfare, Helsinki, Finland; 5 Department of Public Health, University of Helsinki, Helsinki, Finland; 6 Diabetes and Obesity Research Program, University of Helsinki, Helsinki, Finland; 7 Estonian Genome Center, University of Tartu, Tartu, Estonia

## Abstract

The mechanisms through which genes influence body weight are not well understood, but appetite has been implicated as one mediating pathway. Here we use data from two independent population-based Finnish cohorts (4632 adults aged 25–74 years from the DILGOM study and 1231 twin individuals aged 21–26 years from the FinnTwin12 study) to investigate whether two appetitive traits mediate the associations between known obesity-related genetic variants and adiposity. The results from structural equation modelling indicate that the effects of a polygenic risk score (90 obesity-related loci) on measured body mass index and waist circumference are partly mediated through higher levels of uncontrolled eating (β_indirect _= 0.030–0.032, P < 0.001 in DILGOM) and emotional eating (β_indirect _= 0.020–0.022, P < 0.001 in DILGOM and β_indirect _= 0.013–0.015, P = 0.043–0.044 in FinnTwin12). Our findings suggest that genetic predispositions to obesity may partly exert their effects through appetitive traits reflecting lack of control over eating or eating in response to negative emotions. Obesity prevention and treatment studies should examine the impact of targeting these eating behaviours, especially among individuals having a high genetic predisposition to obesity.

The rising prevalence of adiposity is a major public health challenge which is likely to be due to a complex interplay between the obesogenic (i.e. food-rich and sedentary) environment and individual-level factors affecting susceptibility to this environment. Twin studies have shown that a large proportion (47–90%) of within-population variation in body mass index (BMI, kg/m^2^) is attributable to genetic differences between individuals^1^. Genome-wide association (GWAS) studies have increased knowledge of the common genetic variants associated with obesity, and a recent meta-analysis by the GIANT consortium identified 97 BMI-associated single nucleotide polymorphisms (SNPs), of which 56 were novel^2^. Although these loci explained only a small amount (2.7%) of BMI variation, genome-wide estimates have suggested that common genetic variation accounts for 20–30% of the variance in BMI^2^,^3^.

The mechanisms through which genes exert their influence on weight are not well understood. Increased knowledge of these pathways could point to new approaches to obesity prevention and treatment. Many of the common risk variants for obesity are expressed particularly in the hypothalamus, which has a key role in regulating food intake and energy expenditure^2^,^4^; suggesting that traits related to appetite and satiety might represent one behavioural mechanism in the genetic susceptibility to obesity. In line with this hypothesis, quantitative genetic modelling among adult twins has shown that phenotypic correlations between uncontrolled eating (a mixture of items assessing extreme hunger and eating trigged by external food cues), emotional eating (items measuring eating in response to various negative emotions) and BMI are largely due to common underlying genetic influences^5^. These appetitive traits have predicted greater weight gain or weight fluctuations in population-based prospective studies^6^,^7^,^8^,^9^ and therefore are likely to play a causal role in the development of adiposity. Furthermore, there is evidence that children and adults carrying obesity risk alleles, especially in or near the FTO and MC4R genes, have less healthy eating behaviour patterns such as higher energy and fat intakes and higher tendency for snacking^10^,^11^,^12^,^13^,^14^, albeit contrasting results have also been reported^15^,^16^,^17^.

However, only two studies have examined whether appetitive characteristics contribute to explaining the associations between obesity-related genetic variants and anthropometric traits, and both were in children. Wardle *et al.*^18^ and Llewellyn *et al.*^19^ demonstrated that the effects of both the FTO gene and a polygenic risk score (PRS) comprising 28 obesity-related loci on BMI were partly mediated through lower satiety responsiveness. Studies testing these associations in the adult population are needed, since findings on children’s eating behaviour cannot be directly generalized to adults who have more control over their food choices and intakes. A recent study in two cohorts of older US adults^20^ found that a 32-loci PRS was positively related to two appetitive behavioural traits, i.e. uncontrolled and emotional eating, but did not specifically test whether they mediated associations between the genetic variants and adiposity.

The present study aimed to take forward this line of research using data from two independent population-based Finnish cohorts (4632 adults aged 25–74 years and 1231 twin individuals aged 21–26 years). We used structural equation modelling (SEM) to test the hypothesis that the associations between a PRS comprising 90 BMI-related loci and anthropometric traits are partly explained by susceptibility to uncontrolled and emotional eating.

## Methods

### Study cohorts

The DIetary, Lifestyle and Genetic determinants of Obesity and Metabolic syndrome (DILGOM) study was conducted in April-June 2007 and included 5024 Finnish men and women who had participated in the National FINRISK Study in January-March 2007^21^. In FINRISK 2007, a random sample of 10,000 adults (aged 25–74 years), stratified by age and sex, was drawn from the Finnish population register in five geographical areas^22^. This first study phase consisted of a health examination at a municipal health centre and a self-administered health questionnaire. All FINRISK 2007 participants (N = 6258, Response Rate = 63%) were invited to take part in the DILGOM study (N = 5024, Response Rate = 80%). In this second phase, participants completed a shortened and revised Three-Factor Eating Questionnaire (TFEQ-R18)^23^, in the course of the health examination where research nurses measured their weight and waist circumference (WC) and took a blood sample. Genotype data on the SNPs used for the current analyses were available for 4632 DILGOM participants. Information on height was derived from FINRISK 2007, whereas data on all other variables was based on DILGOM.

The FinnTwin12 study^24^ is a cohort of all Finnish twins born in 1983–1987 (N = 2700 families). The first mailed survey was done when the children were 11–12 years (Response Rate = 92%). Data from the fourth wave in 2006–2008, when participants were 21–26 years, were used in the present study. In this wave, 842 individual twins were examined in person in Helsinki and 505 by telephone (total N = 1347, Response Rate = 70%). In the former group, height, weight and WC were measured, and blood samples were taken at the study site for genotyping. The latter group returned saliva DNA kits by mail and self-reported weight and height. All twins completed the TFEQ-R18^23^. Zygosity was determined by well-validated items on physical similarity at school age^25^, and confirmed by genotyping of same-sex twin pairs at the Paternity Testing Unit, National Institute for Health and Welfare, Finland. In the current analyses, information on genome-wide genotypes was available for 1231 individuals. This included 238 monozygotic twin pairs of which only one co-twin was genotyped and the obtained genotype was applied to both co-twins in order to increase statistical power^26^.

### Ethics statement

The research protocols of DILGOM and FinnTwin12 were designed and conducted in accordance with the guidelines of the Declaration of Helsinki. In DILGOM, the protocols were approved by the Ethics Committee of the Hospital District of Helsinki and Uusimaa. In FinnTwin12, the protocols were approved by the Ethics Committee of the Helsinki University Hospital District and the Institutional Review Board of Indiana University. Written informed consent was obtained from all participants in both cohorts.

### Genotyping and weighted PRS

Genotyping of the DILGOM cohort was done at the Wellcome Trust Sanger Institute (Cambridge, UK) and the FIMM Technology Centre (Helsinki, Finland) with the Illumina Cardio-MetaboChip (Illumina, Inc., San Diego, CA, USA)^27^,^28^. To control data quality, sex mismatch and relatedness checks were performed. Thresholds of <95% call rate for each SNP and individual were applied for the genotyped data. The SNPs used in this study were in Hardy-Weinberg equilibrium. FinnTwin12 genotyping was also done at the Wellcome Trust Sanger Institute (Hinxton, UK) on the Human670-QuadCustom Illumina BeadChip (Illumina, Inc., San Diego, CA, USA). Quality control checks and imputation to the 1000 Genomes Phase I integrated variant set release (v3) reference panel have been described previously in Broms *et al.*^29^. The posterior probability threshold for “best-guess” imputed genotypes was 0.9. Genotypes below the threshold were set to missing.

Genetic susceptibility to obesity was assessed by calculating a PRS using 90 of 97 BMI-associated loci identified in the most recent genome-wide meta-analysis^2^. Seven of the 97 SNPs were omitted from the present analyses, four because they were not available on the Cardio-MetaboChip used in DILGOM, and three did not pass the subsequent quality control. The description of the 90 SNPs can be found as Supplementary Table S1. In DILGOM, each genotyped locus could have 0 BMI-increasing alleles, 1 BMI-increasing allele, or 2 BMI-increasing alleles. Altogether, 212 (4.6%) DILGOM participants had missing genotype data on one or more SNPs (mean = 1.6, median = 1, range: 1–9) and missing data for each SNP were imputed using the average coded allele frequency within the cohort^30^. In FinnTwin12, the amount of risk-increasing alleles per locus was 0/1/2 for directly genotyped SNPs and between 0.0 and 2.0 for imputed SNPs. Thus, the potential number of BMI-increasing alleles across the 90 SNPs ranged from 0 to 180 with higher scores indicating a greater genetic predisposition to obesity. A weighted PRS was computed by multiplying the number of BMI-increasing alleles at each locus by its β coefficient with BMI in the European sex-combined analysis derived from the recent meta-analysis^2^.

### Anthropometric traits

Participants’ weight (to the nearest 0.1 kg), height (to the nearest 0.1 cm), and WC (to the nearest 0.5 cm) were measured using standardized international protocols^31^ in both cohorts. Measurements were made in standing position in light clothing and without shoes. WC was measured at a level midway between the lower rib margin and iliac crest. Measured information on BMI (kg/m^2^) and WC was missing for 7 (0.2%) and 29 (0.6%) respondents in DILGOM and for 186 (15.1%) and 296 (24.0%) respondents in FinnTwin12. Only self-reported weight and height were available for FinnTwin12 participants examined by phone. Sensitivity analyses in which missing data on measured BMI were replaced with self-reported data (N = 185) produced highly similar results.

### Appetitive traits

Uncontrolled and emotional eating were assessed with the TFEQ-R18^23^ in both cohorts. The TFEQ-R18 was developed on the basis of a factor analysis of the original 51-item TFEQ in a large sample of Swedish Obese Subjects, and it has been found to be valid in the general population^32^,^33^. The uncontrolled eating scale consists of nine items (e.g., ‘Sometimes when I start eating, I just can’t seem to stop’) and the emotional eating scale three items (e.g., ‘When I feel blue, I often overeat’). Each item was rated on a four-point scale. There were 4348 (93.9%) respondents in DILGOM and 1206 (98.0%) in FinnTwin12 with complete data on uncontrolled and emotional eating items. In the SEM analyses, uncontrolled and emotional eating were modelled as latent factors with the corresponding TFEQ items as indicators. Total scale scores were used in the other analyses and were calculated by averaging the rated items for respondents who had answered at least 5/9 uncontrolled eating items and 2/3 emotional eating items (97.0% in DILGOM and 99.8% in FinnTwin12)^32^.

### Statistical analyses

Age- and sex-adjusted logistic regression and SEM were the main analytical techniques utilized in this study. These analyses were first conducted separately in the two cohorts, and the results were then combined using fixed-effects meta-analysis (between-study heterogeneity was tested with the Q-statistic and in the case of significant heterogeneity [P < 0.001] the combined results should be interpreted with caution). The twin pairs were the primary sampling unit in FinnTwin12 and this clustering of the data was taken into account in all analyses performed in the twin cohort. Logistic regression models were used to quantify how the PRS quartiles (four groups of equal size, see Results for the cut-off points) were associated with the odds of being classified as obese (BMI ≥ 30 kg/m^2^) or abdominally obese (WC ≥ 102 cm in men and WC ≥ 88 cm in women), or being in the highest quartile of uncontrolled or emotional eating (see Results for the cut-off points).

**Figure 1:**
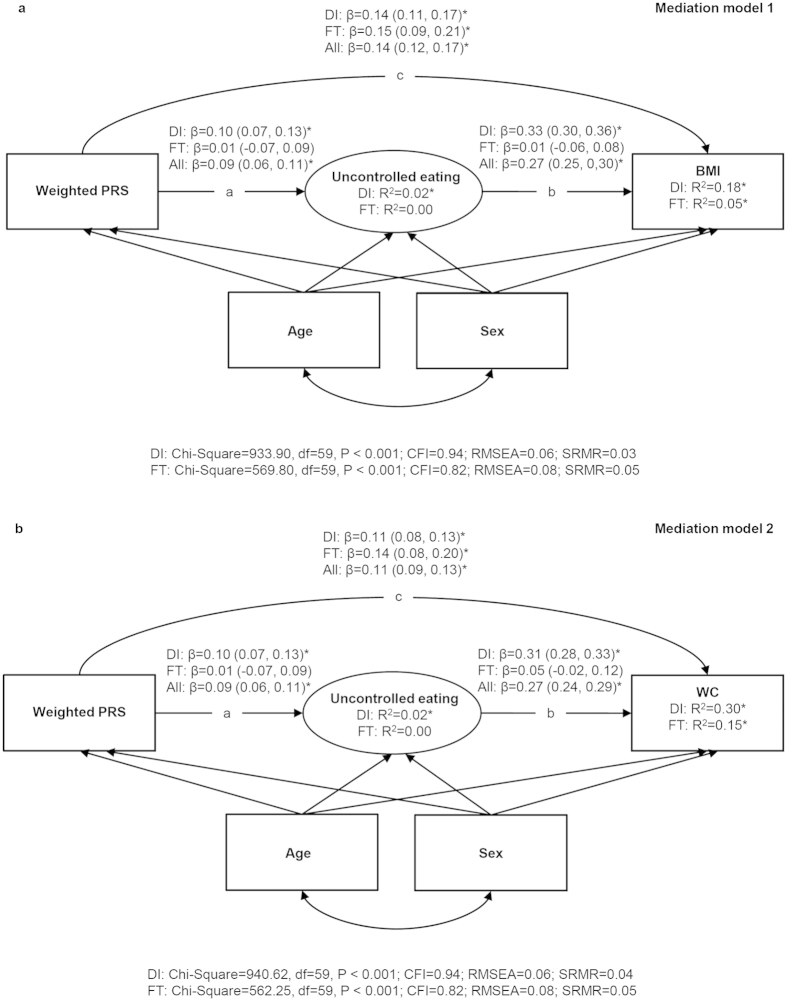
Results from structural equation modelling in the DILGOM (N = 4632) and FinnTwin12 (N = 1231) cohorts and in all participants (meta-analysis). (**a**) Standardized regression coefficients (95% CIs) from the mediation models between the weighted polygenic risk score, uncontrolled eating and BMI. (**b**) Standardized regression coefficients (95% CIs) from the mediation models between the weighted polygenic risk score, uncontrolled eating and WC. Note. All models were adjusted for age and sex in both cohorts and clustering was taken into account in all analyses performed in FinnTwin12. Ellipses represent latent factors (items loading on the latent factors were omitted from the figure for clarity) and rectangles represent observed variables. *P < 0.001; ^†^P < 0.01; ^‡^P < 0.05. PRS = Polygenic risk score; BMI = Body mass index; WC = Waist circumference; DI = DILGOM; FT = FinnTwin12; CFI = Comparative Fit Index; RMSEA = Root Mean Square Error of Approximation; SRMR=Standardized Root Mean Square Residual.

**Figure 2:**
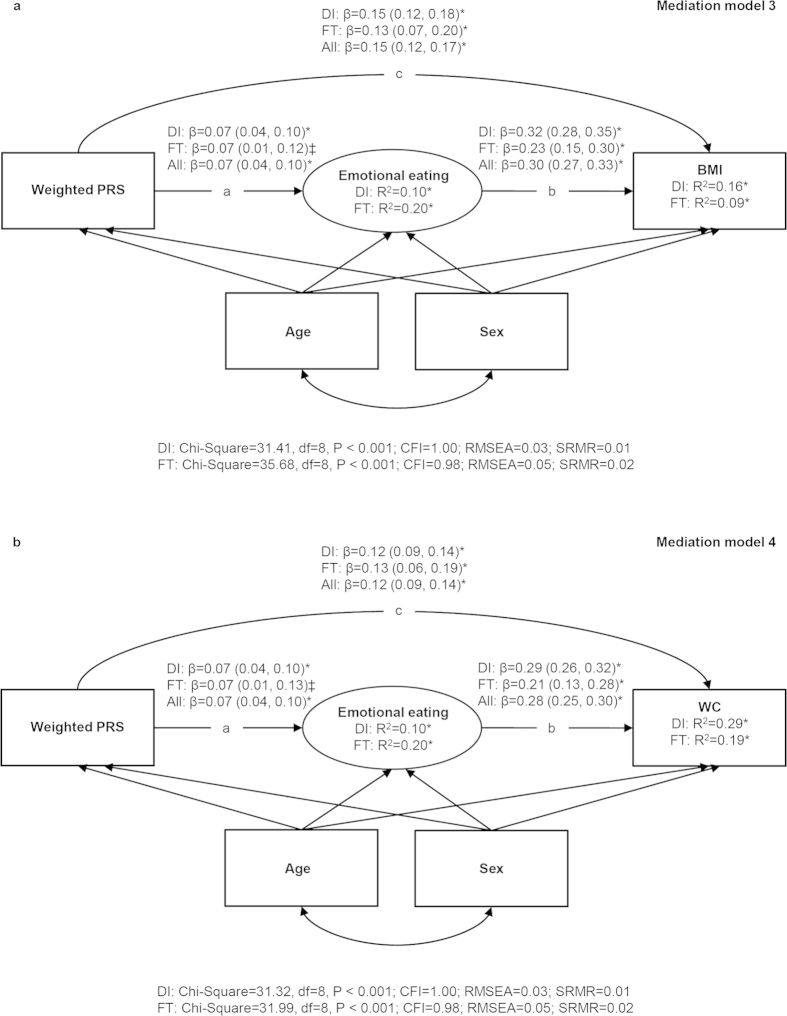
Results from structural equation modelling in the DILGOM (N = 4632) and FinnTwin12 (N = 1231) cohorts and in all participants (meta-analysis). (**a**) Standardized regression coefficients (95% CIs) from the mediation models between the weighted polygenic risk score, emotional eating and BMI. (**b**) Standardized regression coefficients (95% CIs) from the mediation models between the weighted polygenic risk score, emotional eating and WC. Note. All models were adjusted for age and sex in both cohorts and clustering was taken into account in all analyses performed in FinnTwin12. Ellipses represent latent factors (items loading on the latent factors were omitted from the figure for clarity) and rectangles represent observed variables. *P < 0.001; ^†^P < 0.01; ^‡^P < 0.05. PRS = Polygenic risk score; BMI = Body mass index; WC = Waist circumference; DI = DILGOM; FT = FinnTwin12; CFI = Comparative Fit Index; RMSEA = Root Mean Square Error of Approximation; SRMR = Standardized Root Mean Square Residual.

The hypothesized mediation models between the PRS, appetitive traits and adiposity indicators were tested with SEM by using Mplus Version 5^34^. These models were estimated separately for continuous uncontrolled and emotional eating latent factors as well as for continuous BMI and WC variables. The results were reported as the total, direct and indirect effects (via uncontrolled or emotional eating) of the PRS on adiposity indicators (BMI or WC) and their respective 95% CIs. The indirect effect is the product of the direct effects “a” and “b” (Figs 1 and 2) and reflects how much of the association between the PRS and the adiposity indicator is explained by the appetitive trait^35^. The total effect is the sum of the direct effect “c” and indirect effect “ab” (Figs 1 and 2) and represents the relationship between the PRS and the adiposity indicator before adjustment for the appetitive trait. The two-factor structure of the uncontrolled and emotional eating items was initially tested using confirmatory factor analysis. The results largely supported the two-factor structure: the model fit was less optimal in FinnTwin12 (Chi-Square = 564.99, df = 53, P < 0.001; Comparative Fit Index, CFI = 0.87; Standardized Root Mean Square Residual, SRMR = 0.07) than in DILGOM (Chi-Square = 699.14, df = 53, P < 0.001; CFI = 0.96; SRMR = 0.03) based on the recommended cut-off values^36^, but all emotional eating items (λ = 0.79–0.89 in DILGOM and λ = 0.66–0.91 in FinnTwin12) and uncontrolled eating items (λ = 0.46–0.82 and λ = 0.41–0.67, respectively) had reasonably high loadings on their respective factor in both cohorts.

Maximum likelihood estimation with robust standard errors (MLR) was used in the logistic regression and SEM analyses, because the distributions of the study variables deviated from normality to some extent and the observations were non-independent in the twin data^34^. MLR allows estimation with missing data and produces less biased results than conventional techniques (e.g., listwise deletion)^37^,^38^. It does not impute missing values, but estimates parameters and standard errors directly using all the observed data. Descriptive statistics were derived with IBM SPSS Statistics 22 (IBM Corp., Armonk, NY, USA), while logistic regression and SEM were performed using Mplus Version 5 (Muthen & Muthen, Los Angeles, CA, USA) and meta-analysis using Comprehensive Meta-Analysis (Biostat, Englewood, NJ, USA).

## Results

Table 1 displays descriptive characteristics separately for men and women in the two study cohorts. In DILGOM, the average age was 52.7 years. Mean BMI and WC were 27.2 kg/m^2^ and 96.7 cm for men, and 26.8 kg/m^2^ and 86.9 cm for women. Participants in FinnTwin12 had an average age of 22.4 years. Mean BMI and WC were 24.0 kg/m^2^ and 84.5 cm for men, and 22.8 kg/m^2^ and 77.0 cm for women. The weighted PRS had a mean of 2.3 in both cohorts and the cut-off points for the highest PRS quartile were from 2.4 to 2.9 in DILGOM, and 2.4 to 2.8 in FinnTwin12. On a scale from 1 (low) to 4 (high), mean uncontrolled eating scores varied between 1.9 and 2.0, and mean emotional eating scores between 1.5 and 2.1 in the two cohorts. The cut-off scores for the highest uncontrolled eating (from 2.2 to 3.9 in DILGOM, and 2.3 to 4.0 in FinnTwin12) and emotional eating (from 2.3 to 4.0 in DILGOM and in FinnTwin12) quartiles were comparable in both cohorts.

### Polygenic risk for obesity and odds of high BMI, WC and appetite

DILGOM participants in the highest PRS quartile had 2.43 (95% CI 1.97–2.99) higher odds of obesity and 1.93 (95% CI 1.61–2.30) higher odds of abdominal obesity than those in the lowest quartile. The respective odds ratios were 5.42 (95% CI 2.01–14.60) and 1.94 (95% CI 0.99–3.77) in FinnTwin12. DILGOM participants in the highest PRS quartile also had 1.48 (95% CI 1.21–1.80) and 1.44 (95% CI 1.17–1.77) higher odds of high uncontrolled and emotional eating than those in the lowest PRS quartile. Associations between the PRS quartiles and appetitive traits showed a similar pattern in FinnTwin12, but did not reach significance (OR = 1.25, 95% CI 0.84–1.88 for uncontrolled eating; OR = 1.30, 95% CI 0.87–1.93 for emotional eating). When the results were combined using fixed-effects meta-analysis, the odds ratios associated with the highest PRS quartile were 2.51 (95% CI 2.05–3.08) for obesity, 1.93 (95% CI 1.62–2.29) for abdominal obesity, 1.43 (95% CI 1.19–1.71) for high uncontrolled eating, and 1.41 (95% CI 1.17–1.69) for high emotional eating. The Q-statistic indicated that there was no significant between-study heterogeneity in the combined estimates (Q-statistic = 0.00–2.40, P = 0.12–0.99).

### Appetitive traits as mediators in polygenic risk for obesity

Standardized regression coefficients (see Supplementary Table S2 for Pearson’s correlation coefficients between the variables) from the mediation models 1 and 2 (Fig. 1a,b) indicated that DILGOM participants with higher PRS scores had a greater tendency to uncontrolled eating (β = 0.10) and uncontrolled eating was associated with higher BMI (β = 0.33) and higher WC (β = 0.31). The PRS was both directly (β = 0.14 for BMI and β = 0.11 for WC) and indirectly through uncontrolled eating (β = 0.032 for BMI and β = 0.030 for WC) related to greater adiposity (Fig. 1a,b, Table 2). Thus, total associations between the PRS and anthropometric traits were partly mediated by uncontrolled eating in DILGOM. In FinnTwin12, uncontrolled eating was unrelated to the PRS and anthropometric traits and consequently did not mediate the positive total association between the PRS and BMI (β = 0.15) or WC (β = 0.14) (Fig. 1a,b, Table 2). The combined estimates derived from the fixed-effects meta-analysis were consistent with those obtained from DILGOM. Between-study heterogeneity was detected in the effect of uncontrolled eating on BMI (Q-statistic = 69.22, P < 0.001) and WC (Q-statistic = 47.35, P < 0.001), and to a lesser extent in the effect of the PRS on uncontrolled eating (Q-statistic = 3.91, P = 0.048).

Standardized regression coefficients from the mediation model 3 (Fig. 2a) showed that participants scoring higher on the PRS had a slightly greater tendency to emotional eating in DILGOM (β = 0.07) and in FinnTwin12 (β = 0.07). Additionally, emotional eating had a positive association with BMI (β = 0.32 in DILGOM and β = 0.23 in FinnTwin12). The PRS was both directly (β = 0.15 and β = 0.13, respectively) and indirectly through emotional eating (β = 0.022 and β = 0.015, respectively) related to greater BMI (Fig. 2a, Table 2). Thus, total association between the PRS and BMI was partly mediated by emotional eating in both cohorts. Similar findings were obtained with WC as the outcome (Fig. 2b, Table 2). Estimates from the fixed-effects meta-analysis confirmed these results, and only minor between-study heterogeneity was observed in the association between emotional eating and BMI (Q-statistic = 4.93, P = 0.026) and WC (Q-statistic = 4.45, P = 0.035).

### Moderating effect of sex

Multi-group analyses were conducted to test whether the associations between the PRS, appetitive characteristics and adiposity indicators were similar in men and women. In these analyses, the fit of a constrained model (all three path estimates fixed to be the same in the two sexes) was compared with the fit of an unconstrained model (all path estimates allowed to vary by sex) using a chi-square difference test. Non-significant results indicated that the associations did not vary by sex in DILGOM (Δχ^2^ = 2.41–8.50, Δdf = 3, P = 0.037–0.49 with 3/4 P-values > 0.080), or FinnTwin12 (Δχ^2^ = 2.50–6.80, Δdf = 3, P = 0.079–0.48).

## Discussion

This population-based study including both younger and older adults demonstrated that the effects of a 90-loci PRS on both BMI and WC may be partly mediated through higher levels of uncontrolled and emotional eating. These observations offer support to the hypothesis that appetitive traits are one behavioural mechanism through which genes influence adiposity in the current food-rich environment; known as the behavioural susceptibility model^39^.

Our findings add to the emerging body of observational studies demonstrating links between obesity-related genetic variants and phenotypes associated with appetite among children and adults^13^,^18^,^19^,^20^. Two trials in adults found that carriers of risk alleles in the LEP and FTO genes rated their fullness or satiety as lower and hunger as higher after consuming fixed meals or snacks^40^,^41^; providing experimental evidence for the role of these genes in individual differences in feelings of appetite and satiety. Nonetheless, to our knowledge, this is the first study explicitly testing whether appetitive characteristics are intermediate behavioural phenotypes in the polygenic risk for obesity in adults. Llewellyn and colleagues^19^ showed among 10 year-old UK twin children that the effects of a 28-loci PRS on BMI and WC were partly mediated through lower satiety responsiveness. These findings together suggest that appetite is a relevant behavioural pathway in genetic susceptibility to obesity across the life course. As in other studies^32^,^33^, we observed that women scored higher on emotional eating than men (sex differences in uncontrolled eating were less pronounced), but sex did not moderate the associations between the PRS, emotional or uncontrolled eating, and anthropometric traits.

The results were somewhat more consistent in the cohort of 25–74 year-old Finnish adults than in the 21–26 year-old Finnish twins. Uncontrolled eating was not significantly related to the PRS or anthropometric traits in FinnTwin12, but the pattern of results tended to be the same and the smaller sample size (and associated lower statistical power) and the lower prevalence of obesity in the younger FinnTwin12 sample than in DILGOM may partly explain this difference. Overall, the magnitude of the associations between the 90-loci PRS and appetitive traits were small, and only around half of the size of the PRS-adiposity associations. However, phenotypes related to appetite depend on self-report scales and are therefore inherently measured less reliably than adiposity, which may limit the size of the associations with genotype. In addition, there are other appetitive traits, such as satiety responsiveness, which were not included. Many other non-appetitive factors are also likely to be involved in the interplay between genetic variants and body weight. For instance, there is robust evidence that a high level of physical activity attenuates the effects of the FTO gene and other obesity-related variants on BMI^42^,^43^,^44^. Future research could take better account of the multitude of factors when investigating the role of appetite in genetic susceptibility to obesity.

We utilized the TFEQ-R18 to measure appetitive characteristics, and the uncontrolled eating scale contains a mixture of items tapping susceptibility to subjective feelings of hunger, food cravings, and eating triggered by external food cues^23^. However, since items assessing hunger or appetite are dominant in this scale, and confirmatory factor analysis supported one-dimensionality of the scale, uncontrolled eating scores can mainly be considered to reflect subjective feelings of extreme appetite. The TFEQ-R18 includes three items to assess tendency to eat in response to negative emotions. Some researchers^45^ have questioned whether high scores on emotional eating scales capture a real predisposition to eat during negative emotions or whether they reflect concerns about eating or beliefs about the associations between emotions and eating. Nevertheless, the validity of self-reported emotional eating has also received support from experimental studies^46^. We observed a strong correlation between the emotional and uncontrolled eating scales in DILGOM (r = 0.72), while the two scales were more moderately correlated (r = 0.50) in FinnTwin12. This suggests that high scores on the emotional eating scale of the TFEQ-R18 may also reflect general problems in the regulation of eating, particularly in DILGOM participants.

The strengths of the present study included the use of two independent population-based cohorts with identical measurements on appetitive phenotypes and anthropometric traits. It also benefited from the most recent findings on BMI-associated SNPs to construct the PRS, and utilized SEM to test the hypothesized indirect effects. A minor limitation of the study was the use of 90 SNPs instead of the full 97 SNPs identified previously, albeit it is noteworthy that the two risk scores were very highly correlated (r = 0.92) in the twin cohort containing genotype data on all 97 SNPs. A cross-sectional study design is the main limitation and our results need to be confirmed in a longitudinal setting to better ascertain the direction of influences between the polygenic risk for obesity, appetite and adiposity. The expression and action of genes change as a result of aging and in response to environmental exposures. However, since the genome sequence remains the same throughout life, genetic variation is not induced by eating behaviours or changes in weight though gene expression can be influenced by eating behaviours. The interrelationships between appetitive characteristics and anthropometric traits are likely to be complex and bidirectional in nature. In the mediation models, we hypothesized that uncontrolled and emotional eating are determinants of BMI and WC, and not vice versa. A few population-based prospective studies have provided evidence for this by showing that higher initial levels of emotional and disinhibited eating predict greater weight gain over time^6^,^7^,^8^. One study in children explicitly tested whether the prospective association from appetite to weight change was stronger than that from weight to appetite change and did indeed find that the path from appetite to weight was significantly stronger than the other way around^47^.

To conclude, genetic predisposition to obesity may act partly through appetitive traits reflecting lack of control over eating or eating in response to negative emotions. Obesity prevention and treatment studies should seek to identify feasible and effective strategies for improving an individual’s ability to cope with feelings of extreme appetite and the urge to eat in response to external food cues or negative emotions; especially for those with a high genetic susceptibility to obesity. But this is not to deny the importance of the current food-rich environment for obesity risk. The environment offers constant opportunities for overconsumption, ensuring the expression of appetitive traits, driving up food intake for many individuals, and ultimately affecting body weight. Another potential development stems from the increasing availability of personalized genomic information. Preliminary evidence suggests that receiving FTO genetic test feedback can relieve stigma and self-blame related to weight gain, and that it increases readiness to control weight, although to date, there are no studies showing effects on weight^48^,^49^. Research is needed to explore whether the impact of genetic test feedback on behaviour could be enhanced by combining it with strategies that target appetitive characteristics.

## Additional Information

**How to cite this article**: Konttinen, H. *et al.* Appetitive traits as behavioural pathways in genetic susceptibility to obesity: a population-based cross-sectional study. *Sci. Rep.* **5**, 14726; doi: 10.1038/srep14726 (2015).

## Supplementary Material

Supplementary tables

## Figures and Tables

**Table 1:** Descriptive characteristics of the DILGOM and FinnTwin12 cohorts.

	DILGOM (N = 4491–4632)			FinnTwin12 (N = 935–1231)		
	Men/Women	All		Men/Women	All	
	Value	Value	Min-Max	Value	Value	Min-Max
Age (yrs), mean (SD)	53.5 (13.4)/52.0 (13.6)*	52.7 (13.5)†	25–74	22.4 (0.7)/22.4 (0.7)	22.4 (0.7)	21–26
Men, % (N)	–	46.2 (2139)	–	–	46.0 (566)	–
BMI (kg/m^2^), mean (SD)	27.2 (4.1)/26.8 (5.4)*	27.0 (4.8)†	15.8–63.1	24.0 (3.6)/22.8 (3.9)*	23.4 (3.8)	16.4–51.2
Obesity (BMI ≥ 30 kg/m^2^), % (N)	19.6 (418)/22.9 (571)*	21.4 (989)†	–	5.7 (27)/4.7 (27)	5.2 (54)	–
WC (cm), mean (SD)	96.7 (11.9)/86.9 (13.5)*	91.4 (13.7)†	58.0–172.0	84.5 (9.8)/77.0 (9.8)*	80.3 (10.5)	61.0–141.0
Abdominal obesity^a^, % (N)	29.1 (619)/41.1 (1017)*	35.5 (1636)†	–	5.1 (21)/11.9 (62)*	8.9 (83)	–
Uncontrolled eating^b^, mean (SD)	1.85 (0.51)/1.94 (0.54)*	1.90 (0.53)†	1.00–3.89	2.03 (0.53)/2.02 (0.51)	2.02 (0.52)	1.00–4.00
Emotional eating^c^, mean (SD)	1.68 (0.63)/2.10 (0.77)*	1.91 (0.74)†	1.00–4.00	1.48 (0.56)/2.10 (0.78)*	1.81 (0.75)	1.00–4.00
90-loci PRS^d^, mean (SD)	88.7 (6.0)/88.7 (6.1)	88.7 (6.1)	66–111	88.5 (5.7)/89.0 (5.8)	88.8 (5.8)	71–107
Weighted 90-loci PRS^d^, mean (SD)	2.26 (0.16)/2.26 (0.16)	2.26 (0.16)	1.70–2.92	2.26 (0.15)/2.27 (0.15)	2.27 (0.15)	1.84–2.76

Note. N = 2060–2139 for men and N = 2430–2493 for women in DILGOM, and N = 412–566 for men and N = 523–665 for women in FinnTwin12.

^*^Significant (P < 0.05) difference between men and women (ANOVA or chi-square).

^†^Significant (P < 0.05) difference between the two cohorts (ANOVA or chi-square).

^a^WC ≥ 102 cm in men and WC ≥ 88 cm in women.

^b^A mean score of 9 uncontrolled eating items (higher scores indicate greater tendency to uncontrolled eating).

^c^A mean score of 3 emotional eating items (higher scores indicate greater tendency to emotional eating).

^d^Higher scores indicate a greater genetic predisposition to obesity. BMI = Body mass index; WC = Waist circumference; PRS = Polygenic risk score.

**Table 2:** Results from structural equation modelling: total, direct and indirect effects (standardized regression coefficients and 95% CIs) of the weighted polygenic risk score on anthropometric traits.

	DILGOM (N = 4632)	FinnTwin12 (N = 1231)	All (meta-analysis)
	Std. β (95% CI)	Std. β (95% CI)	Std. β (95% CI)
Mediation model 1			
Total effect of weighted PRS on BMI^a^	0.171 (0.143, 0.199)*	0.149 (0.088, 0.210)*	0.167 (0.142, 0.192)*
Direct effect of weighted PRS on BMI^b^	0.139 (0.112, 0.166)*	0.149 (0.088, 0.210)*	0.141 (0.116, 0.166)*
Indirect effect (via uncontrolled eating) of weighted PRS on BMI^c^	0.032 (0.021, 0.043)*	0.000 (−0.001, 0.001)	0.026 (0.016, 0.036)*
Mediation model 2			
Total effect of weighted PRS on WC^a^	0.136 (0.109, 0.162)*	0.139 (0.076, 0.202)*	0.136 (0.113, 0.160)*
Direct effect of weighted PRS on WC^b^	0.106 (0.081, 0.131)*	0.138 (0.075, 0.202)*	0.110 (0.087, 0.134)*
Indirect effect (via uncontrolled eating) of weighted PRS on WC^c^	0.030 (0.020, 0.040)*	0.001 (-0.003, 0.004)	0.026 (0.016, 0.036)*
Mediation model 3			
Total effect of weighted PRS on BMI^a^	0.171 (0.143, 0.199)*	0.149 (0.088, 0.209)*	0.167 (0.142, 0.192)*
Direct effect of weighted PRS on BMI^b^	0.148 (0.121, 0.175)*	0.134 (0.073, 0.195)*	0.146 (0.121, 0.171)*
Indirect effect (via emotional eating) of weighted PRS on BMI^c^	0.022 (0.013, 0.032)*	0.015 (0.000, 0.029)^‡^	0.021 (0.013, 0.029)*
Mediation model 4			
Total effect of weighted PRS on WC^a^	0.135 (0.109, 0.162)*	0.140 (0.077, 0.203)*	0.136 (0.112, 0.159)*
Direct effect of weighted PRS on WC^b^	0.115 (0.090, 0.140)*	0.127 (0.064, 0.190)*	0.117 (0.093, 0.140)*
Indirect effect (via emotional eating) of weighted PRS on WC^c^	0.020 (0.011, 0.029)*	0.013 (0.000, 0.026)^‡^	0.019 (0.011, 0.027)*

Note. All models were adjusted for age and sex in both cohorts and clustering was taken into account in all analyses performed in FinnTwin12. *P < 0.001; ^†^P < 0.01, ^‡^P < 0.05.

^a^Total effect  =  c + ab in Figs 1 and 2;

^b^Direct effect  =  c in Figs 1 and 2;

^c^Indirect effect  =  ab in Figs 1 and 2. PRS = Polygenic risk score; BMI = Body mass index; WC = Waist circumference.
