# Supplementary material for: Appetitive traits as behavioural pathways in genetic susceptibility to obesity: a population-based cross-sectional study
Source: Sci Rep. 2015 Oct 1;5:14726. doi: 10.1038/srep14726 (PMC4589697; doi:10.1038/srep14726)
Supplement: Supplementary tables [file srep14726-s1.pdf]

Appetitive traits as behavioural pathways in genetic susceptibility to obesity: a population-based cross-sectional study

Hanna Konttinen, Clare Llewellyn, Jane Wardle, Karri Silventoinen, Anni Joensuu, Satu Männistö, Veikko Salomaa, Pekka Jousilahti, Jaakko Kaprio, Markus Perola, Ari Haukkala

Supplementary Table S1. Description of the 90 BMI-related SNPs used to calculate the polygenic risk score. Betas and effective alleles are obtained from the European sex-combined analysis from the genome-wide meta-analysis of the GIANT consortium[1]

SNP	Chromosome	Position (hg18)	Position (hg19)	Effect allele	Other allele	β with BMI
rs977747	1	47 457 264	47 684 677	T	G	0.017
rs657452	1	49 362 434	49 589 847	A	G	0.023
rs11583200	1	50 332 407	50 559 820	C	T	0.018
rs3101336	1	72 523 773	72 751 185	C	T	0.033
rs12566985	1	74 774 781	75 002 193	G	A	0.024
rs12401738	1	78 219 349	78 446 761	A	G	0.021
rs11165643	1	96 696 685	96 924 097	T	C	0.022
rs17024393	1	109 956 211	110 154 688	C	T	0.066
rs543874	1	176 156 103	177 889 480	G	A	0.048
rs2820292	1	200 050 910	201 784 287	C	A	0.020
rs13021737	2	622 348	632 348	G	A	0.060
rs10182181	2	25 003 800	25 150 296	G	A	0.031
rs11126666	2	26 782 315	26 928 811	A	G	0.021
rs1016287	2	59 159 129	59 305 625	T	C	0.023
rs11688816	2	62 906 552	63 053 048	G	A	0.017
rs2121279	2	142 759 755	143 043 285	T	C	0.025
rs1460676	2	164 275 935	164 567 689	C	T	0.020
rs1528435	2	181 259 207	181 550 962	T	C	0.018
rs17203016	2	207 963 763	208 255 518	G	A	0.021
rs7599312	2	213 121 476	213 413 231	G	A	0.022
rs492400	2	219 057 996	219 349 752	C	T	0.016
rs2176040	2	226 801 046	227 092 802	A	G	0.014

rs6804842	3	25 081 441	25 106 437	G	A	0.019
rs2365389	3	61 211 502	61 236 462	C	T	0.020
rs13078960	3	85 890 280	85 807 590	G	T	0.030
rs1516725	3	187 306 698	185 824 004	C	T	0.045
rs10938397	4	44 877 284	45 182 527	G	A	0.040
rs13107325	4	103 407 732	103 188 709	T	C	0.048
rs11727676	4	145 878 514	145 659 064	T	C	0.036
rs2112347	5	75 050 998	75 015 242	T	G	0.026
rs7715256	5	153 518 086	153 537 893	G	T	0.016
rs205262	6	34 671 142	34 563 164	G	A	0.022
rs2033529	6	40 456 631	40 348 653	G	A	0.019
rs2207139	6	50 953 449	50 845 490	G	A	0.045
rs9400239	6	109 084 356	108 977 663	C	T	0.019
rs9374842	6	120 227 364	120 185 665	T	C	0.019
rs13201877	6	137 717 234	137 675 541	G	A	0.023
rs13191362	6	162 953 340	163 033 350	A	G	0.028
rs1167827	7	75 001 105	75 163 169	G	A	0.020
rs6465468	7	95 007 450	95 169 514	T	G	0.017
rs17405819	8	76 969 139	76 806 584	T	C	0.022
rs16907751	8	81 538 012	81 375 457	C	T	0.035
rs2033732	8	85 242 264	85 079 709	C	T	0.019
rs4740619	9	15 624 326	15 634 326	T	C	0.018
rs10968576	9	28 404 339	28 414 339	G	A	0.025
rs6477694	9	110 972 163	111 932 342	C	T	0.017
rs1928295	9	119 418 304	120 378 483	T	C	0.019
rs10733682	9	128 500 735	129 460 914	A	G	0.017
rs7899106	10	87 400 884	87 410 904	G	A	0.040
rs17094222	10	102 385 430	102 395 440	C	T	0.025
rs11191560	10	104 859 028	104 869 038	C	T	0.031
rs7903146	10	114 748 339	114 758 349	C	T	0.023
rs4256980	11	8 630 515	8 673 939	G	C	0.021
rs11030104	11	27 641 093	27 684 517	A	G	0.041
rs2176598	11	43 820 854	43 864 278	T	C	0.020
rs3817334	11	47 607 569	47 650 993	T	C	0.026
rs12286929	11	114 527 614	115 022 404	G	A	0.022

rs7138803	12	48 533 735	50 247 468	A	G	0.032
rs11057405	12	121 347 850	122 781 897	G	A	0.031
rs9540493	13	65 103 705	66 205 704	A	G	0.017
rs1441264	13	78 478 920	79 580 919	A	G	0.018
rs10132280	14	24 998 019	25 928 179	C	A	0.023
rs12885454	14	28 806 589	29 736 838	C	A	0.021
rs11847697	14	29 584 863	30 515 112	T	C	0.049
rs7141420	14	78 969 207	79 899 454	T	C	0.024
rs3736485	15	49 535 902	51 748 610	A	G	0.018
rs16951275	15	65 864 222	68 077 168	T	C	0.031
rs7164727	15	70 881 044	73 093 991	T	C	0.018
rs758747	16	3 567 359	3 627 358	T	C	0.023
rs12446632	16	19 842 890	19 935 389	G	A	0.040
rs2650492	16	28 240 912	28 333 411	A	G	0.021
rs3888190	16	28 796 987	28 889 486	A	C	0.031
rs4787491	16	29 922 838	30 015 337	G	A	0.016
rs9925964	16	31 037 396	31 129 895	A	G	0.019
rs2080454	16	47 620 091	49 062 590	C	A	0.017
rs1558902	16	52 361 075	53 803 574	A	T	0.082
rs9914578	17	1 951 886	2 005 136	G	C	0.020
rs1000940	17	5 223 976	5 283 252	G	A	0.019
rs12940622	17	76 230 166	78 615 571	G	A	0.018
rs1808579	18	19 358 886	21 104 888	C	T	0.017
rs7239883	18	38 401 669	40 147 671	G	A	0.016
rs7243357	18	55 034 299	56 883 319	T	G	0.022
rs6567160	18	55 980 115	57 829 135	C	T	0.056
rs17724992	19	18 315 825	18 454 825	A	G	0.019
rs29941	19	39 001 372	34 309 532	G	A	0.018
rs2075650	19	50 087 459	45 395 619	A	G	0.026
rs2287019	19	50 894 012	46 202 172	C	T	0.036
rs3810291	19	52 260 843	47 569 003	A	G	0.028
rs6091540	20	50 521 269	51 087 862	C	T	0.019
rs2836754	21	39 213 610	40 291 740	C	T	0.016

SNP=Single nucleotide polymorphism; BMI=Body mass index.

Supplementary Table S2. Age- and sex-adjusted Pearson's correlation coefficients between the weighted polygenic risk score, appetitive characteristics and anthropometric traits in the DILGOM (N=4632, coefficients are shown above the diagonal) and FinnTwin12 (N=1231, coefficients are shown below the diagonal) cohorts

	Weighted PRS	Uncontrolled eating	Emotional eating	BMI	WC
Weighted PRS	**1.00**	0.10*	0.07*	0.18*	0.15*
Uncontrolled eating[a]	0.01	**1.00**	0.72*	0.35*	0.35*
Emotional eating[a]	0.07†	0.50*	**1.00**	0.32*	0.32*
BMI	0.15*	0.02	0.21*	**1.00**	0.91*
WC	0.15*	0.05	0.19*	0.89*	**1.00**

*P < 0.001; †P < 0.05. [a]Modelled as a latent factor. PRS=Polygenic risk score; BMI=Body mass index; WC=Waist circumference.

References

1. Locke, A. E. et al. Genetic studies of body mass index yield new insights for obesity biology. Nature 518, 197-206 (2015).
